# Effectiveness of Botulinum Toxin Injection With Casting in Children With Spastic Cerebral Palsy: A Randomized Controlled Trial

**DOI:** 10.7759/cureus.61515

**Published:** 2024-06-02

**Authors:** Shivansh Vishwakarma, Dileep Kumar, Ravindra Kumar Garg, Anil K Gupta, Ajai Singh, Sudhir Mishra, Ganesh Yadav

**Affiliations:** 1 Department of Physical Medicine and Rehabilitation, King George's Medical University, Lucknow, IND; 2 Department of Neurology, King George's Medical University, Lucknow, IND; 3 Department of Pediatric Orthopaedics, All India Institute of Medical Sciences, Bhopal, Bhopal, IND

**Keywords:** casting, botulinum toxin injection, equinus, muscle spasticity, cerebral palsy (cp)

## Abstract

Background: The most common form of movement disorder presented in children with cerebral palsy is spasticity, and dynamic equinus is the most common spastic ankle deformity. Botulinum toxin (BT) injection is now an established first-line treatment for focal spasticity.

Aim: To assess the effects of BT injection with casting in the treatment of dynamic equinus in children diagnosed with cerebral palsy with spastic diplegia.

Setting and design: A prospective randomized controlled trial was conducted among patients aged 2-12 years with cerebral palsy and spastic diplegia, attending the general outpatient department and admitted to the indoor facility of the Department of Physical Medicine and Rehabilitation and the Department of Pediatric Orthopedics at King George's Medical University, Lucknow.

Material and methods: Two groups of 19 patients each were formed. Group A received BT injection with casting, whereas in group B, only a cast was applied. Outcome measures including spasticity by Modified Ashworth Scale (MAS), Modified Tardieu Scale (MTS), range of motion (ROM), passive ankle dorsiflexion, and Gross Motor Function Measure (GMFM-66) (dimensions D and E) were assessed before and after the intervention.

Results: The participants in groups A and B were age-matched. A statistically significant difference was seen within group A and group B for MAS, passive ROM-dorsiflexion (PROM-DF), and passive ROM-plantarflexion (PROM-PF) at various follow-ups. In the 3rd week, MAS in each group was statistically insignificant (p-value> 0.05).

Conclusion: There was a significant improvement in tone and a significant increase in the passive range of motion in both groups.

## Introduction

Cerebral palsy (CP) is a neurological disorder that affects development, posture, and movement, leading to a restriction or limitation in the range of motion (ROM) in children as they grow [1]. Various complications occurring in the prenatal, perinatal, and postnatal periods can lead to impairments such as sensory disturbances, perception, motor dysfunctions, affected intelligence, behavioral issues, epilepsy or seizures, and even secondary musculoskeletal problems [2].

The global prevalence of children suffering from CP has been estimated at 1.2% or 12 per 1000 live births [3]. The estimated prevalence of CP in India ranges from 2.1 to 3 per 1000 live births [4].

The most common form of movement disorder presented in children with CP is spasticity [5]. Spasticity is a velocity-dependent resistance to the passive movement of the joint and its associated musculature [6]. It leads to contractures and deformities of bone, ultimately resulting in functional disability. In spastic children, there are positive elements (increased tone, increased deep tendon reflexes, clonus, persistent primitive reflexes) as well as negative elements (decreased coordination, strength, and endurance) [7].

Dynamic equinus is the most common ankle abnormality in children with spastic CP. It presents as an inefficient and unstable gait pattern, and if it is not managed early, it may result in permanent foot deformities [8]. In general, the management of spasticity in children with CP includes oral medications, splinting and casting, physical and occupational therapy, chemo denervation with botulinum toxin (BT) or phenol, intrathecal baclofen administration, selective dorsal rhizotomy, and orthopedic surgery [5,9].

BT injection is now an established first-line treatment for focal spasticity [9]. The most frequent indication for BT type A (BoNT-A) therapy in CP is to treat focal muscle overactivity to improve gait and function in children who can walk. Injection of the upper limb to improve posture and function is the second most frequent indication for BoNT-A therapy in children with CP [9-13].

Another common treatment modality used to combat the spasticity leading to contractures is casting. It is a safe and simple procedure equivalent to other techniques [12]. The number and length of sarcomeres increase after the limb is immobilized in a lengthened position for a sustainable period. Casting has also been seen to increase the ROM [13].

Many studies have assessed the effects of BT injection with casting globally [9-11], but very few studies in India have examined the combined effects of both casting and BT.

This calls for the need for a study to assess the effects of BT injection with casting in the treatment of dynamic equinus in children diagnosed with CP with spastic diplegia.

## Materials and methods

The study was conducted with the approval of ethical clearance from the Institutional Ethics Committee, King George's Medical University, Lucknow (Ref. code: 101st ECM II B-Thesis/P17), and was also registered in the Clinical Trials Registry of India with registration number CTRI/2021/07/035194.

Study setting and participants

A prospective randomized controlled trial was conducted among patients with CP and spastic diplegia, attending the general outpatient department and admitted to the indoor facility of the Department of Physical Medicine and Rehabilitation and from the Department of Pediatric Orthopedics at King George's Medical University, Lucknow, from January 2020 to June 2021. Patients aged 2-12 years, diagnosed with CP with spastic diplegia and normal to mild mental retardation (MR), of Grade 2 or Grade 3 spasticity on the Modified Ashworth Scale (MAS), able to stand or sit with support, and with dynamic equinus deformity were included in the study. Patients with previous surgery to tendo-Achilles or subtalar joint, any contracture of the bilateral lower limb, moderate to profound MR, any systemic illness, allergies to botulinum injection, previous history of phenol or botulinum toxin injection, unwilling patient attendants, local skin lesions, or those on drugs contraindicated with botulinum toxin (e.g., antibiotics such as amikacin, clindamycin, gentamycin, neomycin, tobramycin; anticoagulants like warfarin, heparin; antihistamines; aspirin or other non-steroidal anti-inflammatory drugs; cholinesterase inhibitors; ipratropium, etc.) were excluded from the study.

Sample size

It was calculated using the formula: N = 2(Z(1 - α/2) + Z(1 - β))^2^ SD^2^ /d^2^, where Z(1 - α/2) at 95% confidence interval = 1.96, Z(1 - β) = 0.84 at 80% power, mean difference (d)= 1.85SD, and standard deviation = 2. Sample size (N) = 2 × 2.8 × 2.8 × 4/1.85 × 1.85= 18.44 (approximately 19). Each group had 19 patients. So the total sample size for our study was 38. Two groups were made, each group comprising 19 patients.

Randomization and blinding

All patients fulfilling the inclusion and exclusion criteria were included in the study after obtaining an informed consent form duly signed by each patient’s caregiver. Two groups were made, each comprising 19 patients. Patients were randomized into two groups using a computer-generated randomization table, with an allocation ratio of 1:1. The outcome assessors were blinded in the study. Detailed history and examination were conducted before the intervention.

Intervention

**Figure 1:**
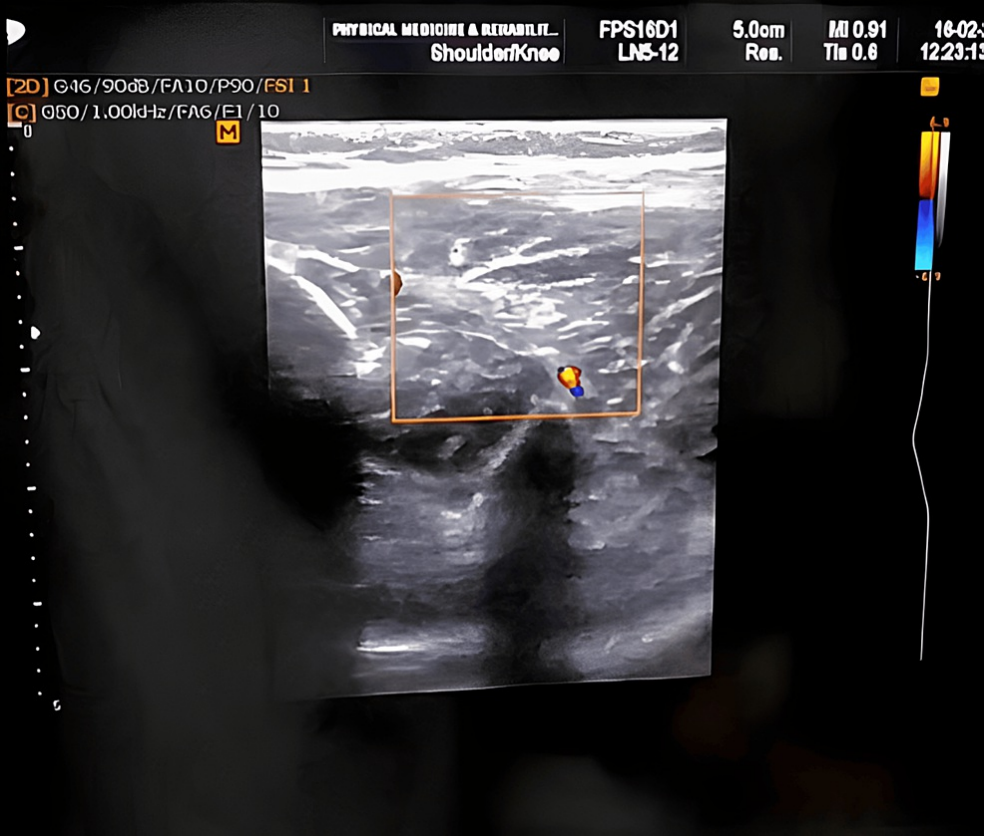
Ultrasound image of the gastrocnemius muscle

**Figure 2:**
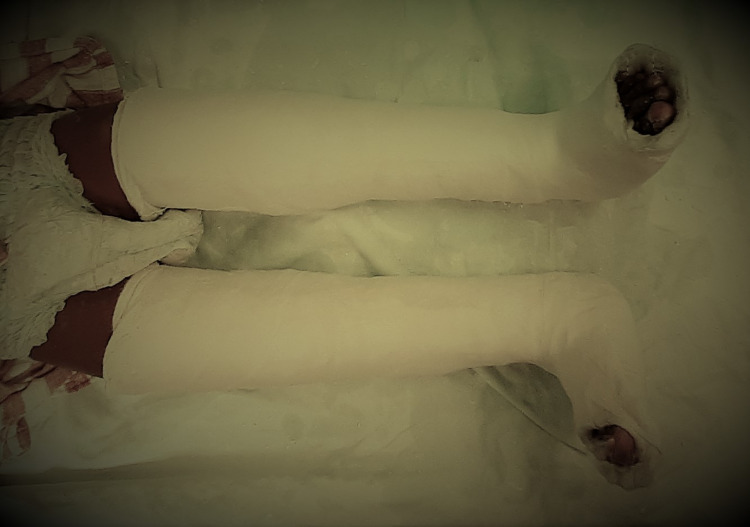
Above-knee cast application in the corrected position

In Group A, botulinum toxin injection was administered under aseptic precautions using ultrasound guidance. The botulinum toxin dose was calculated based on the patient's weight, age, degree of spasticity, and muscle belly size. The muscles targeted for botulinum toxin injection were the medial and lateral heads of the gastrocnemius, soleus, and tibialis posterior (Figure 1). Guidelines for dosing Botox in children included dosing per session as the lesser of 12 U/kg or 400 U, with the dose range being 1-2 U/kg for smaller lower extremity muscles and 3-6 U/kg for larger muscles. No more than 50 U was administered per injection site, and the toxin was diluted with 10 ml of normal saline per 100 U vial. After the botulinum toxin injection, an above-knee cast was applied under general anesthesia to achieve maximum possible dorsiflexion of the foot (Figure 2).

In Group B, an above-knee cast was applied under general anesthesia to achieve maximum possible dorsiflexion of the foot. The range of motion at the ankle joint and the angle in the Modified Tardieu Scale (MTS) were measured using manual goniometry. Both groups received similar exercise programs after the intervention.

The exercise program included 30 minutes of physical therapy daily for six weeks, comprising passive range of motion exercises for the ankle joint, stretching exercises for the plantar flexors, hamstrings, and hip adductor muscle groups, focusing on improving positioning, mobility, and assisting in ambulation.

Outcome

The following parameters were assessed before and after the intervention: Spasticity by MAS, Modified Tardieu Scale (MTS), ROM, passive ankle dorsiflexion, and Gross Motor Function Measure (GMFM-66) (dimensions D and E).

Statistical analysis

Data analysis was performed using IBM SPSS Statistics for Windows, Version 24 (Released 2016; IBM Corp., Armonk, New York). Descriptive analysis was executed for each of the variables using mean, standard deviation, and 95% confidence interval. A p-value of less than 0.05 was considered significant. To evaluate the statistical difference between mean values for observations at different times within the same group, repeated measures ANOVA was used. To evaluate the statistical difference between mean values for different groups, an independent t-test was used. The normality of the data was checked using the Kolmogorov-Smirnov test.

## Results

The participants in groups A and B were age-matched. There was no significant difference between the groups at baseline for the MAS, GMFM-66 Dimension D (%), MTS, passive range of motion-dorsiflexion (PROM-DF), and passive range of motion-plantarflexion (PROM-PF) (p > 0.05) (Table 1).

**Table 1:** Baseline Characteristics of the Study Participants in Both Groups The participants in groups A and B were age-matched. There was no significant difference between the groups at baseline for the Modified Ashworth Scale (MAS), Gross Motor Function Measure (GMFM-66 Dimension D) (%), Modified Tardieu Scale (MTS), passive range of motion-dorsiflexion (PROM-DF), and passive range of motion-plantarflexion (PROM-PF) (p > 0.05). For statistical convenience, MAS grade 1+ was converted to 2, and grades 2, 3, and 4 were converted to 3, 4, and 5, respectively. In MTS, the neutral-null method was used by manual goniometry (positive value for dorsiflexion above neutral and negative value for plantarflexion below neutral at the ankle joint).

Variable	Group A (n=19)	Group B (n=19)	p-value
	Mean ± SD	Mean ± SD	
Age	5.0 ± 2.65	4.47 ± 2.14	0.505
Baseline Modified Ashworth Scale (MAS)	3.58 ± 0.61	3.21 ± 0.63	0.075
Baseline Gross Motor Function Measure (GMFM-66 Dimension D) (%)	58.58 ± 19.04	56.68 ± 14.29	0.731
Modified Tardieu Scale (MTS)			0.209
Positive	2	5	
Negative	17	14	
Passive range of motion-dorsiflexion (PROM-DF)			0.349
0	6	4	
0-5	6	3	
0-10	7	11	
0-20	0	1	
Passive range of motion-plantarflexion (PROM-PF)			0.276
0-40	16	12	
0-45	2	3	
0-50	1	4	
0-55	0	0	

The mean MAS in group A was 3.58 ± 0.61 at baseline, 1.79 ± 0.54 at the 3rd week, and 1.63 ± 0.50 at the 6th week. In group B, the mean MAS was 3.21 ± 0.63 at baseline, 2.16 ± 0.60 at the 3rd week, and 2.21 ± 0.53 at the 6th week. In both groups, the changes over time were statistically significant. Similarly, a significant difference was observed within both group A and group B for PROM (DF) and PROM (PF) at various follow-ups (Table 2).

**Table 2:** Intra-group Comparison of Outcome Measures at Various Follow-ups The mean MAS in group A was 3.58 ± 0.61 at baseline, 1.79 ± 0.54 at the 3rd week, and 1.63 ± 0.50 at the 6th week. In group B, the mean MAS was 3.21 ± 0.63 at baseline, 2.16 ± 0.60 at the 3rd week, and 2.21 ± 0.53 at the 6th week. The changes in MAS were statistically significant in both groups. Similarly, a significant difference was observed within both group A and group B for PROM (DF) and PROM (PF) at various follow-ups.

Outcome		Baseline	3^rd^ week	6^th^ week	p-value
Modified Ashworth Scale (MAS)	Group A	3.58 ± 0.61	1.79 ± 0.54	1.63 ± 0.50	0.0001
	Group B	3.21 ± 0.63	2.16 ± 0.60	2.21 ± 0.53	0.0001
Passive range of motion-dorsiflexion (PROM-DF)	Group A	1.05 ± 0.85	2.42 ± 0.61	2.63 ± 0.50	0.0001
	Group B	1.47 ± 0.91	1.68 ± 0.67	2.11 ± 0.73	0.042
Passive range of motion-plantarflexion (PROM-PF)	Group A	0.211 ± 0.54	2.26 ± 0.56	2.42 ± 0.51	0.0001
	Group B	0.579 ± 0.84	1.68 ± 0.48	2.00 ± 0.00	0.0001
Gross Motor Function Measure (GMFM 66, Dimension D) (%)	Group A	58.58 ± 19.04	58.74 ± 19.11	58.83 ± 19.13	0.999
	Group B	56.68 ± 14.29	56.79 ± 14.34	56.83 ± 14.32	0.919
GMFM 66 (Dimension E)	Group A	23.32 ± 10.54	23.47 ± 10.50	23.48 ± 10.51	0.989
	Group B	23.47 ± 14.01	23.53 ± 14.01	23.57 ± 14.06	1.001

In the 3rd week, the mean MAS in group A was 1.79 ± 0.54, and in group B, it was 2.16 ± 0.60, but this difference was not statistically significant. However, by the 6th week, the mean MAS was 1.63 ± 0.50 in group A and 2.21 ± 0.53 in group B, and this difference was statistically significant. No difference was observed in the other outcome parameters between the groups, indicating an equivocal effect of both treatment modalities (Table 3).

**Table 3:** Inter-group Comparison of Outcome Measures at Various Follow-ups In the 3rd week, the mean MAS in group A was 1.79 ± 0.54, and in group B, it was 2.16 ± 0.60, with no statistically significant difference between the groups. However, by the 6th week, the mean MAS was 1.63 ± 0.50 in group A and 2.21 ± 0.53 in group B, and this difference was statistically significant. No difference was observed in the other outcome parameters between the groups, indicating the equivocal effect of both treatment modalities.

Follow-ups	Outcome	Group A	Group B	p-value
3^rd^ week	Modified Ashworth Scale (MAS)	1.79±0.54	2.16±0.60	0.050
	Gross Motor Function Measure (GMFM-66, Dimension D) (%)	59.53±19.44	56.79±14.34	0.724
	GMFM-66 (Dimension E) (%)	23.63±10.54	23.53±14.01	0.717
6^th^ week	MAS	1.63 ± 0.50	2.21 ± 0.53	<0.01
	GMFM-66 (Dimension D) (%)	62.16 ± 18.80	58.21 ± 14.96	0.990
	GMFM-66 (Dimension E) (%)	24.21 ± 10.25	24.42 ± 14.29	0.982

## Discussion

Our present study confirms the effectiveness of BT A injection with casting in the treatment of dynamic equinus in spastic CP children. Both groups experienced significant short-term improvements in terms of spasticity measurement (MAS), MTS, and PROM for dorsiflexion and plantarflexion. As the follow-up period of the study was limited to six weeks, it is difficult to comment on the long-term sustainability of the improvements from casting and BT injection. A greater change in GMFM-66 score in dimension D than in dimension E was observed in the group receiving BT A along with casting, but the results were not statistically significant.

The results of the study indicate a clear benefit from BT injection in treating dynamic equinus deformity in spastic CP children. There were more significant and marked changes in the parameters assessed at baseline, 3rd week, and 6th week in the group receiving BT injection along with casting compared to the group receiving casting alone.

The mean age of the children in groups A and B was 5.0 ± 2.65 years and 4.47 ± 2.14 years, respectively, which is similar to a study by Kay et al. [14], where the mean age was 6.9 ± 2.8 years in group A and 7.3 ± 3.3 years in group B. In assessing spasticity, the parameter used was the Modified Ashworth Scale (MAS), where it was found that the baseline MAS was 3.58 ± 0.61 in group A and 3.21 ± 0.63 in group B. This is comparable to the study by Kay et al. [14], where the baseline MAS was 2.6 ± 1.2 in group A and 2.6 ± 1.1 in group B.

In this study, changes in the MAS were observed after the 3rd and 6th weeks post-intervention. In group A, the MAS was 1.79 ± 0.54 (p = 0.093) and 1.63 ± 0.50 (p < 0.01), respectively, while in group B, it was 2.16 ± 0.60 (p < 0.01) and 2.21 ± 0.53 (p < 0.01), respectively. In a study conducted by Park et al. [15], a 1-month post-intervention assessment showed a statistically significant reduction in MAS to 1.5 ± 0.7 (p < 0.05). Another study by Kay et al. [14] had a 12-week follow-up similar to our study. At 12 weeks, the reduction in MAS was statistically significant in both groups (p = 0.0122 in Group A and p = 0.0031 in Group B). Another study by Kelly et al. [16] found changes in MAS after 1, 2, and 6 months post-intervention in group A were 1.3 ± 0.5, 1.5 ± 0.5, and 2.0 ± 0.8, respectively (p = 0.001). Similar significant reductions were seen in a study by Lee et al. [17], where MAS after the 3rd, 6th, and 12th weeks post-intervention was 1.3 ± 0.3, 1.2 ± 0.7, and 1.2 ± 0.3, respectively (p < 0.001). Therefore, there is a significant improvement (reduction in spasticity) in both groups, but more marked changes are seen in group A receiving BT injection along with casting than in group B receiving casting alone.

In assessing gross motor functions after the intervention in both groups, changes in GMFM-66 dimension D scores after the 3rd and 6th weeks post-intervention in group A were 58.74 ± 19.11 (p = 0.083) and 58.83 ± 19.13 (p = 0.058), respectively, and in group B, the scores were 56.79 ± 14.34 (p = 0.163) and 56.83 ± 14.32 (p = 0.07), respectively. For dimension E, the scores after the 3rd and 6th weeks post-intervention in group A were 23.47 ± 10.50 (p = 0.083) and 23.48 ± 10.51 (p = 0.064), respectively, and in group B, the scores were 23.53 ± 14.01 (p = 0.331) and 23.57 ± 14.06 (p = 0.09), respectively.

The study by Park et al. [15] found changes in GMFM-66 dimension D scores one month post-intervention in group A to be 79.49 ± 26.60 (p < 0.05), while dimension E scores in group A were 65.60 ± 32.96. Another study by Kay et al. [14] reported changes in GMFM-66 dimension C, D, and E scores three months post-intervention to be 2.5 ± 7.5 (p = 0.2853) in group A and -1.3 ± 5.1 (p = 0.8105) in group B. Additionally, a study by Dai et al. [18] found changes in GMFM-66 scores in the group receiving serial casting after BTX-A injection at the 6th and 12th weeks to be 69.42 ± 5.29 (p < 0.001) and 77.37 ± 6.81 (p < 0.001), respectively.

Our study shows that improvement in GMFM-66 dimension D scores after the 3rd and 6th weeks post-intervention in group A (BTX-A with casting) is numerically better than in group B (cast alone). These results are comparable to the study by Park et al. [15]. However, changes in dimension E were not noteworthy in any group, as the activities are more difficult to achieve in dimension E than in dimension D. The study by Bottos et al. [19] shows significant improvement in dimension D (p = 0.052) and dimension E (p = 0.007) in the group receiving BTX-A with a single cast after four months post-intervention. This suggests that dimension E can improve with long-term assessment. The activities in dimension E are much more complex than those in dimension D, which likely require more time to show improvement, as evidenced by long-term follow-up in many studies.

The limitation of our study was that, due to the nature of the intervention, allocation concealment and blinding of the participants were not possible, which might have affected the outcome. Additionally, the short follow-up period in the study also impacted the results.

## Conclusions

The results of the present study show desirable effects from both groups. There was a significant improvement in tone (reduction in spasticity grade as measured by MAS and MTS), a significant increase in passive range of motion (both dorsiflexion and plantarflexion), and more marked changes in GMFM-66 scores of dimensions D and E in group A, which received BT A injection along with casting. This suggests that the use of BT can yield better results when combined with casting rather than casting alone.
